# Association Between Perinasal Uptake on Radioactive Iodine Whole-Body Scan and Nasolacrimal Duct Obstruction

**DOI:** 10.3390/medicina61071165

**Published:** 2025-06-27

**Authors:** Minjung Seo, Hongje Lee, Na Ri Park, Ju-Hyang Lee, Seol Hoon Park

**Affiliations:** 1 Department of Nuclear Medicine, Ulsan University Hospital, University of Ulsan College of Medicine, Ulsan 05505, Republic of Korea; 0733285@uuh.ulsan.kr; 2 Department of Nuclear Medicine, Dongnam Institution of Radiological & Medical Sciences (DIRAMS), Busan 46033, Republic of Korea; drhjlee@outlook.com; 3 Department of Opthalmology, Ulsan University Hospital, Ulsan 44033, Republic of Korea; 4 Namu Eye Clinic, Yongin-si 16841, Republic of Korea

**Keywords:** nasolacrimal duct obstruction, radioactive iodine, whole body scan, dacryoscintigraphy

## Abstract

*Background and Objectives:* This study reports an association between nasolacrimal duct obstruction (NLDO) and perinasal uptake on radioactive iodine (RAI) whole-body scan. *Materials and Methods*: This is a retrospective study of 37 patients from May to November 2017 who underwent thyroidectomy and I-131 ablation for papillary thyroid cancer (PTC) and had a follow-up I-123 diagnostic WBS and dacryoscintigraphy. Ophthalmic examinations assessed punctal stenosis, NLDO, tear film break-up time, Schirmer’s test, punctate keratopathy, tear meniscus height, epiphora, and ocular dryness. Perinasal and nasal uptake on whole-body scans (WBSs) were assessed as negative (no uptake) or positive (focal uptake). The associations between perinasal uptake on WBS, dacryoscintigraphy findings, and ophthalmic assessments were assessed. *Results*: Nasal uptake on I-131 post-ablation WBS were observed in 60 eyes (81%); perinasal uptake was observed in 8 eyes (11%). Nasal uptake on I-123 post-ablation WBS were observed in all eyes; perinasal uptake was observed in 15 eyes (20%). Perinasal and nasal uptake on I-131 post-ablation WBS were significantly associated with delayed excretion on dacryoscintigraphy (*p* < 0.001 and *p* = 0.03, respectively). Perinasal uptake on I-123 WBS was associated with both abnormal dacryoscintigraphy findings and ocular dryness (*p* < 0.001 and *p* = 0.02, respectively). *Conclusions*: Perinasal uptake on I-131 post-ablation and I-123 diagnostic WBS was significantly associated with delayed excretion on dacryoscintigraphy, suggesting NLDO.

## 1. Introduction

Radioactive iodine (RAI) therapy is widely used for remnant ablation or as adjuvant treatment in patients with differentiated thyroid cancer (DTC) following thyroidectomy [1]. It improves survival and reduces recurrence in these patients [2,3,4]. After administration, iodine-131 (I-131) is actively transported into thyroid follicular or tumor cells via the sodium–iodide symporter (NIS) [1,5]. However, the NIS is also expressed in various extrathyroidal tissues such as the salivary glands, gastric mucosa, breast, thymus, lacrimal gland, and nasolacrimal drainage system [6]. Unintended irradiation of these tissues during RAI therapy can cause side effects including nasolacrimal duct obstruction (NLDO) [7,8].

Although salivary and lacrimal gland dysfunctions after I-131 therapy are well documented, studies focusing specifically on NLDO are limited [9,10]. A significant proportion of ingested radioactive iodine is excreted in tears, potentially leading to the damage of the lacrimal gland and nasolacrimal duct [11,12]. NLDO is a well-established complication of I-131 therapy, with reported incidences ranging from 2% to 18%, particularly in patients over 45 years of age or those receiving cumulative doses above 100–150 mCi (3.7–5.55 GBq) [13,14,15]. The pathogenesis involves radiotoxic epithelial injury mediated by the NIS, leading to inflammation, fibrosis, and obstruction of the lacrimal drainage system [14,16]. Morphological studies confirm epithelial desquamation, mucous gland injury, and fibrosis [16]. The American Thyroid Association and other reports stress the importance of clinician awareness, patient counseling, and timely referral for dacryocystorhinostomy when indicated [1,7,17]. To date, no study has directly assessed whether the abnormal I-131 uptake on WBS reflects functional nasolacrimal duct obstruction as confirmed by dacryoscintigraphy.

Current clinical practice mainly relies on symptom-based monitoring. In this study, we aimed to determine whether perinasal RAI uptake on WBS is associated with the dysfunction of the lacrimal drainage system, which could serve as an early predictor of lacrimal excretory dysfunction, as confirmed by dacryoscintigraphy.

## 2. Methods

### 2.1. Study Subjects

This study retrospectively analyzed 37 thyroid cancer patients who underwent thyroidectomy and I-131 ablation at Ulsan University Hospital (UUH) between May and November 2017, and then underwent a follow-up I-123 diagnostic WBS, dacryoscintigraphy, and ophthalmic examinations. The institutional review board at UUH approved the retrospective use of clinical data for this study (IRB No. 2023-3-015).

### 2.2. Data Analysis

#### 2.2.1. I-131 and I-123 Whole-Body Scans

Patients underwent post-ablation I-131 WBS on either the 2nd or 7th day following RAI administration. I-123 WBSs were performed at least 100 days post-therapy. Perinasal and nasal uptake on WBS was classified as either negative (no uptake) or positive (focal uptake). Two nuclear medicine physicians reached a consensus on scan interpretations.

#### 2.2.2. Lacrimal Drainage System

##### A. Dacryoscintigraphy

Dacryoscintigraphy was performed in a seated position, while ensuring that the SPECT detector was positioned as close to the eye as possible. A single drop of technetium-99m pertechnetate (50–100 µCi/eye) was instilled in the outer canthus of each eye. Images were acquired using SPECT with a high-resolution collimator. Delayed tracer excretion through the nasolacrimal system on dacryoscintigraphy was considered abnormal. Two nuclear medicine specialists reached a final interpretation by consensus.

##### B. Ophthalmic Examination

An ophthalmologist assessed patients’ lacrimal drainage system function using various tests such as punctal stenosis, NLDO, tear film break-up time (BUT), Schirmer’s test, punctate keratopathy, tear meniscus height (TMH), epiphora, and ocular dryness. NLDO was graded from 0 to 3: 0, none; 1, (1–50% reflux); 2, (50–99% reflux); and 3, total obstruction. An abnormal BUT was defined as a duration of <10 s. An abnormal Schirmer’s test was defined as tear production of <10 mm in 5 min. TMH was graded as 1, low; 2, low-to-normal; 3, normal; and 4, increased.

### 2.3. Statistical Analysis

Fisher’s exact test or chi-square test was used to assess the concordance between perinasal uptake on WBS and findings from the dacryoscintigraphy or ophthalmic assessment. SPSS version 21 (IBM SPSS Statistics Software, Armonk, NY, USA) was used for statistical analysis, with *p* < 0.05 considered statistically significant.

## 3. Results

### 3.1. Patient Characteristics and Examination Results

In total, 74 eyes (10 male; mean age of 50 years.) were studied. The mean RAI dose was 75 mCi, with 62% of patients receiving more than 30 mCi. No significant demographic differences were observed between patients receiving low (≤30 mCi) and high (>30 mCi) RAI doses. On post-ablation I-131 WBS, ipsilateral perinasal uptake was observed in 11% of patients and nasal uptake in 81%. On I-123 diagnostic WBS, perinasal and nasal uptake rates were 20% and 100%, respectively. The RAI ablation dose was not significantly associated with perinasal or nasal uptake in WBS. Abnormal dacryoscintigraphy findings were observed in 32% of patients.

On ophthalmic examination, NLDO (16%), ocular dryness 41%, epiphora (15%), and punctal stenosis (12%) were present. The demographic characteristics and detailed study results are presented in Table 1.

### 3.2. Association Between WBS and Dacryoscintigraphy

Eyes with ipsilateral perinasal uptake on I-131 and I-123 WBSs exhibited abnormal dacryoscintigraphy findings in 8 out of 8 eyes and 11 out of 15 eyes, respectively. Nasal uptake on I-131 and I-123 WBSs showed abnormal dacryoscintigraphy results of 38% (23/60 eyes) and 32% (24/50 eyes), respectively. A statistically significant association was found between perinasal uptake on I-131 WBS and abnormal dacryoscintigraphy findings (*p* < 0.001). Nasal uptake also showed a statistically significant correlation with abnormal dacryoscintigraphy findings (*p* = 0.03).

**Figure 1:**
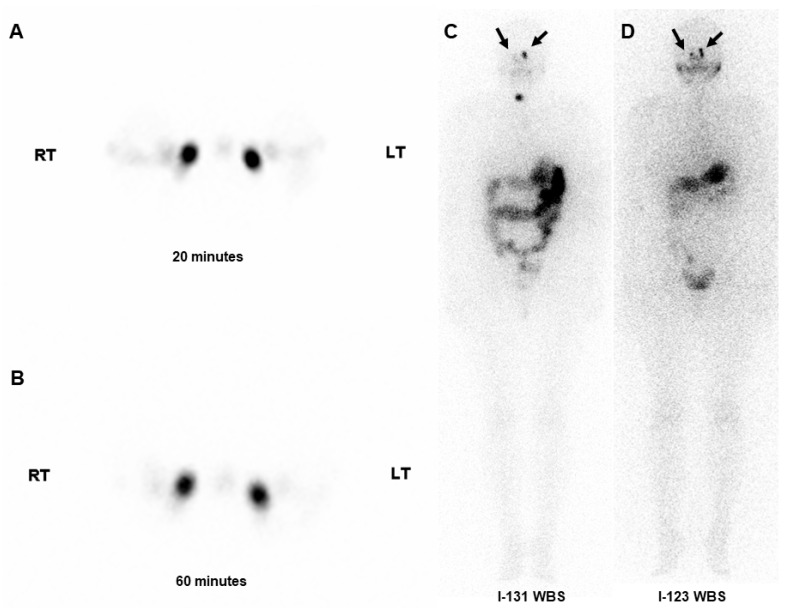
A patient who underwent radioactive iodine ablation exhibited a severe excretion delay on early (**A**) and late (**B**) images of dacryoscintigraphy. Although nasal uptake was relatively minimal, bilateral perinasal uptakes (arrows) were observed on both I-131 (**C**) and I-123 whole-body scans (**D**). Remnant thyroid uptake was also noted on I-131 whole-body scan.

Similarly, perinasal uptake on I-123 WBS was significantly associated with abnormal dacryoscintigraphy findings (*p* < 0.001). Nasal uptake was not statistically evaluated. No statistically significant association was found between perinasal uptake on I-131 WBS and ophthalmic examinations, and nasal uptake was also not statistically significant. However, perinasal uptake on I-123 WBS was significantly associated with ocular dryness (*p* = 0.02), whereas no significant associations were identified with other ophthalmic parameters. Nasal uptake on I-123 WBS was not significantly correlated with any ophthalmic examination findings. The associations between WBSs and abnormal lacrimal system findings, as evaluated using dacryoscintigraphy and ophthalmic examinations, are summarized in Table 2. A representative case of abnormal dacryoscintigraphy and WBS findings is shown in Figure 1. The patient exhibited bilateral perinasal uptake on both I-131 and I-123 WBSs, whereas nasal uptake was relatively minimal. Although the ophthalmic examination was unremarkable, dacryoscintigraphy showed a severe excretion delay.

## 4. Discussion

We show that perinasal uptake on I-131 and I-123 WBSs are significantly associated with abnormal dacryoscintigraphy findings. To our knowledge, this is the first study to examine the relationship between extrathyroidal uptake on RAI WBSs and dacryoscintigraphy. Several reports have suggested that nasolacrimal duct or nasal uptake is linked to lacrimal dysfunction [18,19], but none have confirmed this using dacryoscintigraphy, which is a minimally invasive imaging test widely used to evaluate the function of the lacrimal excretory system and to diagnose NLDO. Dacryoscintigraphy allows dynamic assessment of tear excretion and is more sensitive in detecting partial or early-stage obstructions than anatomical imaging or lacrimal irrigation [18].

Bakheet et al. showed that radioactivity in tears collected via Schirmer’s test peaked 1 h after the ingestion of 15 mCi of I-123 and remained detectable at 24 h [12]. In this study, I-131 and I-123 WBSs were obtained on the 7th day and 24 h post-ingestion, respectively. Under normal conditions, the lacrimal glands, tear film, and lacrimal excretory system are not seen on RAI WBSs due to their small size, low tear flow rate, and rapid turnover rate of the tear film. However, individuals who exhibited radioactivity in these regions likely had a higher concentration of RAI uptake in the lacrimal excretory system, with prolonged retention compared to those without uptake. Such patients may be at an increased risk of NLD toxicity, as suggested by the findings of this study.

This study examined the association between the RAI WBS findings and ophthalmic examination. Although prior studies have reported RAI-related dacryocystitis, epiphora, and tear production changes, no study has comprehensively assessed the lacrimal excretion system via ophthalmologic evaluation. Only perinasal uptake on I-123 WBS was significantly associated with ocular dryness, while no significant associations were found with other ophthalmic parameters. However, dacryoscintigraphy successfully identified lacrimal excretion abnormalities that ophthalmologic examinations did not detect. Although NLDO is a recognized complication of RAI therapy, clinical management typically remains symptom driven. Patients are advised to monitor for tearing or ocular symptoms, which often arise 6 to 12 months following treatment [14,15]. However, identifying early imaging markers could enhance risk stratification, personalize follow-up protocols, and improve patient education. Asymptomatic individuals exhibiting perinasal uptake on WBSs may benefit from further evaluation with dacryoscintigraphy to detect early or subclinical dysfunction. Extended follow-up periods would be helpful to confirm this hypothesis.

This study also evaluated both perinasal and nasal uptake on WBSs. Several studies reported nasal uptake and described nasolacrimal duct uptake on post-ablation WBS [19,20]; however, no study has systematically analyzed uptake at both sites using diagnostic WBSs. In this study, perinasal uptake on I-131 WBS exhibited a stronger association with abnormal dacryoscintigraphy findings than that on I-123 WBS. Nasal uptake was highly prevalent (81% on I-131 and 100% on I-123 WBSs). Based on these findings, perinasal uptake on RAI WBSs may serve as a useful predictor of lacrimal excretory system abnormalities. This study had several limitations, including its small size and lack of follow-up clinical and imaging data. Larger scale studies with longer follow-up durations would be helpful to confirm our study.

## 5. Conclusions

Perinasal uptake on I-131 post-ablation and I-123 diagnostic WBSs was significantly associated with delayed excretion on dacryoscintigraphy, suggestive of NLDO. Perinasal uptake may serve as a predictive marker of preclinical lacrimal excretory dysfunction and support the need for close surveillance and ophthalmological follow-up.

## Figures and Tables

**Table 1:** Patient characteristics, radioactive iodine whole-body scan findings, dacryoscintigraphy results, and ophthalmic examination outcomes in 37 patients (74 eyes).

Variable	Results
Sex, n	
Men	10
Women	27
Mean age, years (range)	50 (22–74)
Mean RAI dose, mCi (range)	75 (30–150)
Abnormal RAI WBS uptakes	
Perinasal uptake (+), % (eyes)	
I-131 WBS	11 (8)
I-123 WBS	20 (15)
Nasal uptake (+), % (eyes)	
I-131 WBS	8 (60)
I-123 WBS	100 (74)
Abnormal dacryoscintigraphy, % (eyes)	32 (24)
Abnormal ophthalmic examination, % (eyes)	
Nasolacrimal duct obstruction	16 (12)
Ocular dryness	41 (30)
Epiphora	15 (11)
Punctal stenosis	12 (9)
Tear film break-up time	96 (71) *
Schirmer’s test	25 (18/72) ^†^
Punctate keratopathy	51 (38)
Tear meniscus height	35 (26)

n, number; RAI, radioactive iodine; WBS, whole-body scan. * Schirmer’s test was not performed in one patient (two eyes). ^†^ Tear film break-up time was not performed in one patient (two eyes).

**Table 2:** Association between radioactive iodine whole-body scan findings and lacrimal duct system assessments.

Variable	I-131 WBS						I-123 WBS					
	Perinasal Uptake			Nasal Uptake			Perinasal Uptake			Nasal Uptake		
	No	Yes	p *	No	Yes	p *	No	Yes	p *	No	Yes	p *
Dacryoscintigraphy			<0.001			0.03			<0.001			NA
Normal	50	0		13	37		46	4		0	50	
Abnormal	16	8		1	23		13	11		0	24	
Punctal stenosis			0.58			0.36			1.0			NA
No	57	8		11	54		52	13		0	65	
Yes	9	0		3	6		7	3		0	9	
NLDO			1.0			0.44			0.44			NA
No	55	7		13	49		48	14		0	62	
Yes	11	1		1	11		11	1		0	12	
Epiphora			1.0			0.11			0.22			NA
No	56	7		14	49		52	11		0	63	
Yes	10	1		0	11		7	4		0	11	
Dryness			0.13			0.16			0.02			NA
No	37	7		6	38		31	13		0	44	
Yes	29	1		8	22		28	2		0	30	
Tear film break-up time			1.0			0.18			1.0			NA
Normal	7	1		3	5		7	1		0	5	
Abnormal	57	7		11	53		50	14		0	64	
Schirmer’s test			0.67			0.74			0.1			NA
Normal	17	1		4	14		17	1		0	18	
Abnormal	47	7		10	44		40	14		0	54	
Punctate keratopathy			0.15			0.91			0.68			NA
Normal	30	6		7	29		28	8		0	36	
Abnormal	36	2		7	31		31	7		0	38	
Tear meniscus height			1.0			1.0			1.0			NA
Normal	65	8		14	59		58	15		0	73	
Abnormal	1	0		0	1		1	0		0	1	

WBS, whole-body scan; NA, not assessable; NLDO, nasolacrimal duct obstruction (assessed by an ophthalmologist). * Chi-square or Fisher’s Exact Test.

## Data Availability

No new data were created or analyzed in this study. Data sharing is not applicable to this article.
